# Imaging in Cutaneous Melanoma: Current Workup, Surveillance, and Emerging Directions

**DOI:** 10.3390/cancers18142215

**Published:** 2026-07-09

**Authors:** Haley Willem, Tyler Aguilar, Arthur W. Cowman, Kristel Lourdault, Richard Essner

**Affiliations:** Saint John’s Cancer Institute, Providence Health & Services, Division of Surgical Oncology, Borstein Family Melanoma Program, Laboratory of Cutaneous Oncology, Santa Monica, CA 90404, USA; haley.willem@providence.org (H.W.); tyler.aguilar@providence.org (T.A.); acowman@hs.uci.edu (A.W.C.); kristel.lourdault@providence.org (K.L.)

**Keywords:** melanoma, radiographic imaging, cutaneous lesions, sentinel lymph node biopsy, artificial intelligence, staging, computed tomography, positron emission tomography

## Abstract

Imaging techniques used for the care of cutaneous melanoma patients have greatly changed over the past century, from symptom-driven radiography toward a multimodality framework integrated for staging, directing surgery, and systemic therapy, and surveillance. Historically, clinical evaluation and skin exams have been the tenets of melanoma diagnosis and staging. In recent years, noninvasive imaging, such as dermoscopy, total-body photography and reflectance confocal microscopy, has expanded the diagnostic toolset for primary melanoma detection. Concurrently, several imaging techniques have been developed to detect metastases and follow disease progression, including computed tomography (CT), magnetic resonance imaging (MRI), fluorodeoxyglucose positron emission tomography/computed tomography (FDG PET/CT), lymphoscintigraphy, and single-photon emission computed tomography/computed tomography (SPECT/CT). The use of immune checkpoint inhibitors has also altered imaging interpretation by introducing atypical response patterns, including pseudoprogression, requiring immune-adapted assessment frameworks such as Immune Response Evaluation Criteria in Solid Tumors (iRECIST). While there is a strong consensus for high-risk patients, imaging techniques and surveillance schedules for low-risk patients (stage I/II) remain controversial due to limited supporting evidence and conflicting data on costs and patient benefit. The development of new technologies, including image-guided surgery, non-FDG PET tracers, phone apps, artificial intelligence-assisted image analysis, and radiomics, may further change melanoma imaging. The aim of this review is to detail the historical evolution of melanoma imaging, the development of new imaging techniques, and their role and future in clinical practice.

## Simple Summary

Diagnosis and treatment of cutaneous melanoma rely heavily on imaging technologies, which have evolved over time. Historical techniques include clinical evaluation and skin examination, while modern modalities such as ultrasound, radioactive tracer-directed imaging, computed tomography (CT) and magnetic resonance imaging (MRI) provide more accurate visualization of disease. These advancements in imaging techniques have allowed for greater accuracy in detecting primary tumors, lymph node metastases and distant metastases. These improvements may lead to better treatment planning for melanoma patients through earlier detection and more accurate staging. Imaging techniques continue to evolve, particularly with the development of new radioactive tracers that may provide more precise and specific imaging and the use of artificial intelligence to efficiently analyze these images. This review provides a comprehensive overview of imaging techniques used for the care of melanoma patients, dating from the historical era through modern and technological advancements, with a focus on the clinical utility of these techniques.

## 1. Introduction

Melanoma practice in the United States generally follows the American Joint Committee on Cancer (AJCC) 8th edition staging framework, which stratifies disease risk according to tumor thickness, ulceration status, mitotic rate of the primary tumor, nodal status and the presence of distant metastases [1,2,3]. Early-stage melanoma is often curable with surgical excision, but the risk of recurrence rises substantially with increasing tumor thickness, ulceration, nodal involvement, and related pathologic features [1,4,5]. While clinicopathologic variables remain the foundation of staging and management, imaging provides noninvasive evaluation of disease status, guiding surgery, systemic therapy and surveillance.

Over the past several decades, the role of imaging in melanoma has expanded from a limited confirmatory tool for clinically apparent metastatic disease to a central component of disease management by informing initial staging, surgical planning, treatment response assessment, and post-treatment surveillance [6,7]. This shift has occurred in parallel with major therapeutic advances. Immune checkpoint inhibitors and targeted therapies have improved patient outcomes in metastatic melanoma, increasing the importance of imaging for baseline staging, early detection of recurrence and nuanced interpretation of imaging findings that could greatly inform treatment decisions [8,9]. Simultaneously, immunotherapy has made image interpretation more complex, particularly when inflammatory changes due to treatment mimic progression, requiring different response criteria [10].

Accordingly, melanoma imaging is best understood as a dynamic component of long-term disease management, integrated with clinical decision-making and evolving therapeutic strategies. This review aims to describe the historical evolution of cutaneous melanoma imaging, outlining current clinical practice across disease staging, treatment response, metastasis detection, treatment decision-making and surveillance. Additionally, we discuss future directions to provide insight into where imaging techniques are heading, especially with the use of artificial intelligence (AI). Because most melanoma cases are of the cutaneous subtype, this review does not cover ocular or other melanoma subtypes. The aim of this review is to describe the historical evolution of melanoma imaging, outline current clinical practice across staging, treatment response assessment, and surveillance, and discuss emerging technologies and their potential to redefine the field of imaging.

## 2. Search Methods

Literature research was conducted through established databases, primarily PubMed, Embase, and Google Scholar. Search space was restricted based on year (1980–2026, with emphasis on studies published after 2010 except for landmark studies published earlier) and subject (studies has to include melanoma patients or models, unless the paper described an imaging technique broadly or there was no literature on melanoma for the modality). Because of the temporal breadth of this review, covering imaging applications in melanoma care from the historical through the modern era, publication years were not heavily restricted.

Only primary research articles, book chapters, and meta-analysis articles were included; research letters or other unique article types were excluded. Review articles were used extremely sparingly, primarily to provide technical explanations of widely used imaging modalities or technologies. All search terms included “cutaneous melanoma,” except for studies explaining fundamental theories of imaging modalities. Some recently developed imaging modalities or imaging-based technologies do not yet have abundant research on cutaneous melanoma but are still highly relevant. In these cases, the terms “melanoma,” or “skin cancer” were also used to broaden search criteria to similar conditions.

For identifying literature pertaining to specific imaging techniques, the name of the modality was included in the search term. For identifying literature on performance or efficacy, the terms “accuracy,” “sensitivity,” “specificity,” “feasibility,” or “performance” were included. The AI sections used the search terms “AI” or “artificial intelligence”.

## 3. Radiographic Imaging in Melanoma

### 3.1. Brief History of Melanoma Imaging

For much of the twentieth century, melanoma staging relied primarily on clinical examination and histopathologic assessment of the skin and lymph nodes. Imaging was reserved for patients with suspected metastatic disease and was limited in sensitivity for early systemic spread. Chest radiography was widely used but demonstrated poor performance for detecting small pulmonary metastases [11,12]. A major conceptual advance emerged in the late 1980s with the introduction of sentinel lymph node biopsy (SLNB), enabled by lymphoscintigraphy mapping [13,14]. This technique improved detection of occult regional lymph nodal metastases while reducing morbidity compared to elective lymph node dissection, and by the early 2000s became incorporated into melanoma staging systems [13,14]. Lymphatic mapping represented one of the earliest examples of imaging directly guiding melanoma management.

The adoption of cross-sectional imaging, beginning in the 1990s and accelerating through the early 2000s, further expanded staging capabilities. Computed tomography (CT) improved detection of thoracic and abdominal metastases, and magnetic resonance imaging (MRI) became the preferred modality for brain metastasis detection [15,16]. The integration of fluorodeoxyglucose positron emission tomography (FDG PET), followed by hybrid FDG PET/CT systems, became widely adopted for surveillance in patients with advanced-stage melanoma [17,18]. More recently, hybrid approaches such as single-photon emission computed tomography/computed tomography (SPECT/CT), have further refined pre-operative nodal localization, particularly in anatomically complex regions [19]. Together, these imaging advancements have established the foundation for the AJCC 8th edition [1], and continued prospective work refining molecular staging parameters may alter some imaging thresholds in the coming years.

### 3.2. Imaging for Initial Staging and Risk Stratification

Current imaging strategies in melanoma are strongly stage- and risk-adapted, reflecting recommendations from the National Comprehensive Cancer Network (NCCN) and the European Society for Medical Oncology (ESMO) [6,7]. Patients with stage I and stage IIA melanoma generally do not undergo routine systemic imaging due to the low likelihood of occult distant metastasis and the risk of false-positive findings [6,7]. In contrast, patients with higher-risk disease, particularly stage IIB/IIC and III, are commonly considered for baseline imaging to evaluate subclinical metastatic disease that would alter management [6,7].

**Figure 1:**
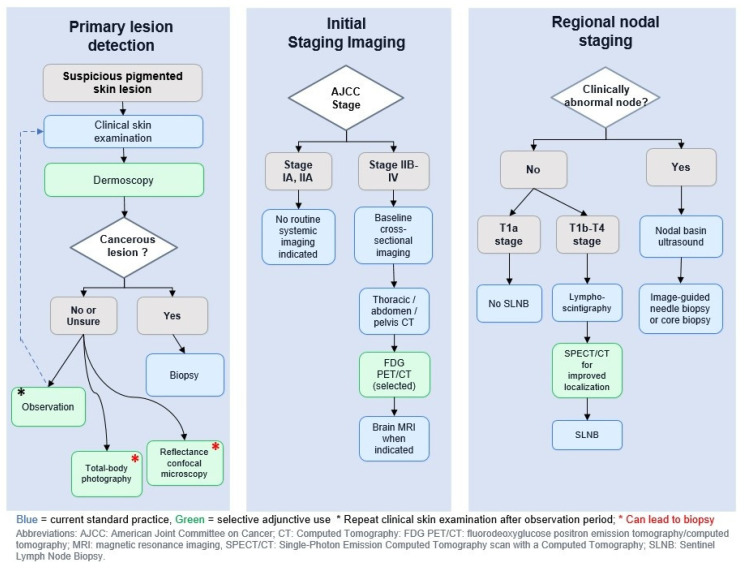
Clinical imaging pathway in cutaneous melanoma for primary lesion detection, initial staging, and regional nodal staging. Blue boxes represent current standard practice, green boxes represent selective adjunctive, * (black) shows that clinical skin examination is repeated after the observation period and * (red) shows that the TBP and RCM results can lead to biopsy. Note: AJCC: American Joint Committee on Cancer; CT: computed tomography; FDG PET/CT: fluorodeoxyglucose positron emission tomography/computed tomography; MRI: magnetic resonance imaging, SPECT/CT: single-photon emission computed tomography scan with computed tomography; SLNB: sentinel lymph node biopsy.

For patients with advanced-stage melanoma, cross-sectional imaging with CT or FDG PET/CT is typically used for systemic evaluation. However, FDG PET/CT does not replace SLNB, as it lacks sufficient spatial resolution to detect microscopic nodal metastases [11,14]. The increasing availability of systemic therapies with intracranial activity has strengthened the rationale for early brain imaging in high-risk populations, particularly with MRI [20]. Lastly, ultrasound serves a complementary role in the evaluation of regional lymph nodes (LNs) and is frequently used to guide biopsy of suspicious nodal disease [11]. Overall, modern staging relies on a multimodal strategy combining surgical staging with cross-sectional and functional imaging (Figure 1).

### 3.3. Imaging for Treatment Response Assessment

The assessment of treatment response in melanoma has become increasingly complex with the use of modern immunotherapy. Conventional size-based criteria, such as Response Evaluation Criteria in Solid Tumors (RECIST 1.1), remain widely used but have several limitations in this setting [21]. Immune checkpoint inhibitors can produce atypical response patterns, including transient tumor enlargement or the appearance of new lesions followed by subsequent regression, a phenomenon known as pseudoprogression [22,23]. To address these challenges, immune-adapted criteria such as immune RECIST, or iRECIST, introduced the concept of unconfirmed progression and recommend repeat imaging before declaring treatment failure in clinically stable patients [10]. iRECIST defines “unconfirmed progression” as a first assessment showing new lesions or more than a 20% increase in tumor burden, without clinical deterioration, that must be confirmed on repeat imaging four to eight weeks after the “unconfirmed progression” observation and before any treatment decisions are made [10]. In practice, this means a stable or improving patient with a concerning scan typically warrants a short-interval follow-up rather than immediate treatment discontinuation. However, when radiographic progression is associated with clinical deterioration, treatment modification should be decided without waiting for confirmatory imaging [10,24].

**Figure 2:**
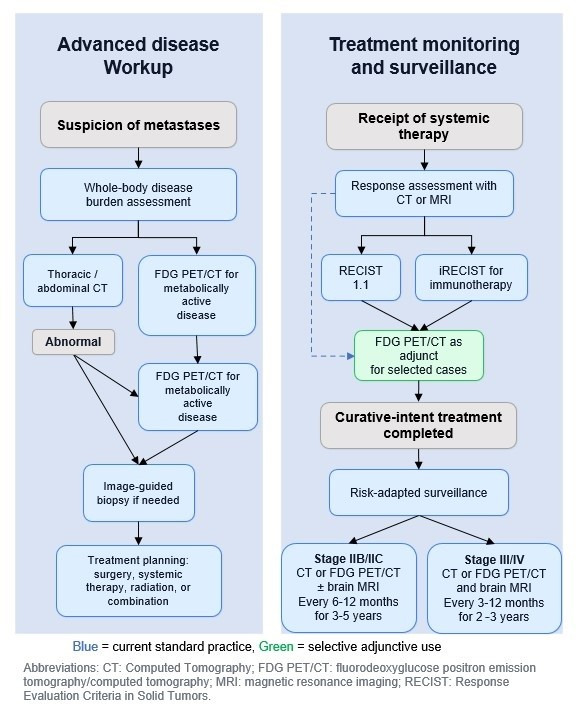
Clinical imaging pathway in cutaneous melanoma for treatment monitoring, surveillance, and advanced metastatic disease workup. Blue boxes represent current standard practice and green boxes represent selective adjunctive tools. Note: CT: computed tomography; FDG PET/CT: fluorodeoxyglucose positron emission tomography/computed tomography; MRI: magnetic resonance imaging; RECIST: Response Evaluation Criteria in Solid Tumors.

Functional imaging with FDG PET/CT may provide additional insight into treatment response, as metabolic changes can appear before measurable tumor shrinkage can be visualized [25]. Accordingly, response assessment increasingly relies on longitudinal imaging integrated with clinical status, rather than rigid reliance on size-based criteria alone (Figure 2). However, immune-related inflammatory uptake can complicate interpretation, particularly in patients receiving checkpoint inhibitors [25].

### 3.4. Surveillance Imaging Schedules in Melanoma Follow-Up

Post-treatment surveillance strategies in melanoma remain heterogeneous and continue to evolve. Historically, follow-up relied primarily on clinical examination, with imaging reserved for symptomatic patients [11]. The availability of effective new systemic therapies has increased the clinical need for early detection of recurrent disease [8,9,26,27]. Surveillance imaging depends on the patient’s risk to minimize over-scanning while guaranteeing metastatic detection. Patients with stage I and IIA melanoma are generally followed with regular skin examinations without routine imaging, whereas patients with stage IIB–IV disease are often considered for routine imaging, with frequency varying based on the patient’s risk level [6,7]. In practice, CT or FDG PET/CT is commonly used for whole-body surveillance, while MRI is employed for intracranial imaging in patients at high risk for brain metastases. Commonly, imaging is done every three to twelve months during the first two to three years for stage III/IV patients, while it is done every six to twelve months during the first three to five years for stage IIB/IIC patients [16,28]. Despite widespread use, there is limited evidence that routine surveillance imaging improves overall survival, and its primary value may lie in enabling earlier clinically actionable disease [16,28].

There are consequences to excessive surveillance, and these should be part of shared decision-making. False-positive rates with CT and FDG PET/CT are not trivial. A meta-analysis of prospective FDG PET/CT studies in cutaneous melanoma found pooled false-positive rates of 5.8% [95% CI, 3.3%–8.8%] on a patient-based level and 9.1% [95% CI, 3.4%–17.2%] on a lesion-based level [29]. When evaluating the performance of FDG PET-CT on melanoma distant metastasis, a reported positive predictive value of 78% [95% CI, 0.70–0.84], revealing that false-positive findings are particularly frequent among patients undergoing routine surveillance without clinical suspicion of relapse [30]. Each suspicious finding requires further workup, carries diagnostic risk if biopsy is pursued, and induces patient anxiety that may persist well beyond its resolution [29,31]. A cohort study of asymptomatic stage III patients found that 7% of all patients had false-positive PET findings and 6% underwent biopsy to determine this, showcasing a meaningful burden in a surveillance population [31]. The cost-effectiveness of intensive surveillance regimens in moderate-risk populations has not been rigorously established. Modeling work from Dieng et al., estimated that 12-monthly (=once a year) PET/CT surveillance in resected stage III disease carried an incremental cost-effectiveness ratio of AUD (Australian Dollar) 34,362 per additional distant recurrence correctly diagnosed and treated compared with no surveillance imaging—a threshold that, while within accepted bounds, tightens considerably with more frequent scanning intervals and has not been validated for the stage IIB/IIC population, in whom routine imaging remains controversial [32].

International guidelines are not uniform. A study reviewing NCCN, ESMO, the British Association of Dermatologists, and the German S3 imaging guidelines for high-risk melanoma follow-up found no consensus on imaging modality, staging-specific thresholds, or recommended surveillance intervals. Some organizations do not recommend routine imaging while others do, especially CT or PET/CT, but without specifying frequency [16]. This lack of alignment extends even to the two most influential guidelines: the European one declines to define a surveillance frequency, offering only an example schedule and justifying it by the absence of comparative evidence favoring any regimen, whereas the NCCN recommends stage-stratified imaging schedules [7,33]. Another study comparing guidelines showed that surveillance with PET/-CT or CT was recommended by 15 guidelines and MRI by 11, most commonly in stage IIC or higher disease, though with variable frequency and total duration [34]. Clinicians should consult the current version of the applicable guideline and engage patients in discussion of individual risk, expected yield, relevant costs and the tradeoffs involved.

While imaging can detect asymptomatic disease and facilitate earlier treatment, it is also associated with false positives, increased cost, and patient anxiety [16,28]. Consequently, current guidelines emphasize individualized surveillance strategies based on recurrence risk and clinical context [6,7]. Emerging approaches using tumor or blood biomarkers may further refine surveillance methods and complement imaging in the detection of recurrence [35,36,37]. Instead of indiscriminately ordering testing, future melanoma surveillance will likely depend on integration of imaging with other molecular biomarkers to estimate the risk of recurrence. This approach aims for high detection of disease while minimizing unnecessary imaging. As systemic therapies improve, optimizing surveillance strategies will remain critical to balancing early detection with costs, patient morbidity, and clinical benefits (Figure 2).

### 3.5. Radiomics in Melanoma Imaging

Radiomics refers to the high-throughput extraction and analysis of quantitative features from medical images. By encoding tumor shape, intensity distribution, and textural heterogeneity from standard medical images, this visual data is converted into mineable numerical representations [38]. The assumption of radiomics is that phenotypic information encoded within imaging exceeds subjective visual assessment. Regions of intra-tumoral heterogeneity reflecting hypoxia, necrosis, differential proliferation, and microenvironmental variation may produce measurable signal—at the voxel level—before those features are clinically apparent [39]. In melanoma specifically, this holds promise given the well-documented biological heterogeneity of advanced disease and the ongoing challenge of predicting immunotherapy response at the individual patient level.

The standard radiomic pipeline involves image acquisition, lesion segmentation (manually, semi-automated, or increasingly deep-learning-assisted), feature extraction using established software libraries, dimensionality reduction, and model training and validating for clinical endpoints [38]. Feature extraction typically proceeds in three dimensions; 3D volumetric analysis is now substantially more common than 2D approaches across published studies, reflecting a consensus that volumetric methods better capture lesion heterogeneity. CT scans have been the imaging modality most commonly used, appearing in more than 40% of melanoma radiomic studies published to date, followed by MRI and FDG PET/CT—a distribution that reflects both the central role of CT in routine melanoma staging and the relative availability of annotated CT datasets for model development [40].

The integration of radiomics into FDG PET/CT has extended the prognostic vocabulary beyond conventional semiquantitative parameters. Radiomics-derived metabolic tumor volume (MTV) and total lesion glycolysis (TLG) are promising imaging biomarkers, whereas standardized uptake value (SUV) is not, as they capture only scalar summaries of lesion-level FDG avidity. Radiomic approaches have been developed to characterize intra-tumoral heterogeneity by extracting textural features from PET data—a meaningful extension given that cutaneous melanoma exhibits high biological heterogeneity, including hypoxic areas, necrotic regions, zones of high cellular proliferation, and intra-tumoral angiogenic variation [41]. Total-body MTV and TLG have emerged as independent prognostic factors for survival in advanced melanoma. Although no validated cut-off value for patient stratification has been established in clinical practice [41], this reinforces that quantitative PET metrics remain research tools rather than guideline-endorsed decision thresholds.

The most clinically relevant application of radiomics in melanoma to date has been in predicting response to immune checkpoint inhibitor therapy—the setting in which the limitations of RECIST-based assessment are most accurate [42,43,44]. Several studies have reported encouraging discriminative performance. For example, Dercle et al. developed a radiomic model predicting six-month OS in patients with advanced melanoma treated with anti-PD-1 therapy, achieving an AUC of 0.82 and outperforming conventional RECIST-based assessments [43]. Brendlin et al. demonstrated that CT dual-energy radiomics could improve prediction of immunotherapy response in stage IV melanoma, particularly through assessment of lesion-specific heterogeneity, as investigators found that lesions with higher structural heterogeneity were 1.7 times less likely to be non-responders than patients with higher lesion homogeneity [44]. Delta-radiomics computes feature differences between pre- and post-treatment imaging rather than relying on baseline scans alone. These features enable early identification of responders. One pilot study created a model to predict early response to immunotherapy using pre- and post-treatment CT scans and delta features. The model achieved an AUC of 0.882 in the training dataset and 0.857 in the validation cohort, and it demonstrated potential to distinguish pseudoprogression from true progression [45].

The appeal of radiomics for pseudoprogression discrimination is intuitive. Textural features may capture immune-driven changes in lesion composition before clinical presentation. They could help clinicians avoid premature treatment discontinuation in the patients most likely to eventually respond. Preliminary work has explored the prediction of hyperprogression at both the lesion and patient levels using FDG PET±CT-based radiomic models. In a cohort of 56 consecutive metastatic melanoma patients treated with immune checkpoint inhibitors, 330 metastatic lesions were individually segmented on pre-treatment CT and FDG PET imaging, and CT-, PET-, and combined PET/CT-based models were built to predict lesion hyperprogression at three months. The CT-based model achieved an AUC of 0.760 and 0.703 in the training and testing datasets, respectively. The combined PET/CT model performed similarly (training AUC, 0.756; testing AUC, 0.704), whereas the PET-only model failed to validate, with a training AUC of 0.629 and a testing AUC of 0.516 [46]. This addresses a clinical question of particular urgency, given that hyperprogression under checkpoint inhibition carries a substantially worse prognosis than conventional disease progression.

Despite individually promising findings, the melanoma radiomic literature is characterized by heterogeneity of results and a lack of validation. Several studies, including those by Ter Maat et al. and Peisen et al., found that radiomic features alone or in combination with clinical data did not significantly improve prognostic prediction over traditional clinical models [47,48]. The Peisen study specifically examined whether whole-body baseline CT radiomics combined with clinical parameters outperformed clinical parameters alone in predicting three-month response and six- and twelve-month OS in stage IV melanoma patients receiving checkpoint inhibitor therapy. They reported a potential but non-significant added value of radiomics for survival prediction, underscoring the ongoing relevance of established clinical parameters. Using CT scans from 262 stage IV melanoma patients, they created a model that did not significantly outperform the one using clinical features only, showing AUCs of 0.664 [95% CI, 0.598–0.729] vs. 0.620 [0.545–0.692] and 0.600 [0.526–0.667] vs. 0.588 [0.481–0.629] for six- and twelve-month overall survival, respectively [47]. In this cohort of 262 stage IV melanoma patients, all visible metastases were three-dimensionally segmented, and the extended model (clinical parameters plus radiomics) did not significantly outperform the baseline clinical-only model for overall survival at six months (AUC 0.664 [95% CI, 0.598–0.729] vs. 0.620 [0.545–0.692]) or twelve months (0.600 [0.526–0.667] vs. 0.588 [0.481–0.629]), nor for response prediction at three months (0.641 [0.581–0.700] vs. 0.656 [0.587–0.719]). Similarly, Ter Maat et al. concluded that baseline CT-derived radiomics are moderately predictive of checkpoint inhibitor outcomes in advanced melanoma but do not improve upon simpler clinical predictors, and that combining radiomics with clinical variables does not yield meaningfully better predictions, attributing this finding to substantial overlap in the information captured by both model types. In their multicenter cohort of 620 patients, of whom 59.2% experienced clinical benefit, the radiomics model achieved an AUROC of 0.607 [95% CI, 0.562–0.652], lower than that of the clinical model (0.646 [0.600–0.692]), and the combined model did not improve discrimination (0.636 [0.592–0.680]), with the difference between the ensemble and clinical models not reaching statistical significance [48].

A 2025 systematic review and meta-analysis encompassing 40 studies and 4673 patients provides the most comprehensive synthesis currently available [40]. Across 24 studies investigating treatment response and survival prediction, a random-effects model estimated a pooled AUC of 0.83 [95% CI: 0.74–0.92], indicating strong overall discriminative performance of the included radiomic models, with low to moderate heterogeneity (I^2^ = 28.6%) [40]. However, this must be interpreted cautiously as it reflects a literature skewed toward smaller, single-center, retrospective studies with a risk of overfitting, and external validation is often absent.

The principal barrier to clinical translation remains reproducibility. Radiomics can quantify image-derived tumor heterogeneity and support diagnosis, prognosis, and treatment assessment, yet adoption has been limited by poor reproducibility across scanners, protocols, and software [49,50]. Variations in imaging protocols, scanner types, tumor segmentation methods, and feature extraction techniques can introduce inconsistency and reduce reproducibility in ways that are difficult to detect when studies are conducted within a single institution using uniform acquisition parameters [40]. The Image Biomarker Standardization Initiative (IBSI), established in 2015 and completing its second phase in 2024, has worked to harmonize feature definitions and preprocessing pipelines across compliant software platforms [49,50]. IBSI compliance has meaningfully improved consistency of feature extraction, but challenges related to image acquisition protocols and preprocessing steps continue to limit comparability across studies [49]. Segmentation variability, whether performed manually by a radiologist or by automated algorithms with differing boundary definitions, remains a particularly consequential source of feature instability that no computational standard fully resolves.

The near-term trajectory of radiomic research in melanoma points toward integration rather than standalone applications. The most promising models combine radiomic features with clinical variables (stage, LDH, and ECOG performance status), molecular biomarkers (BRAF/NRAS status and tumor mutational burden), and, increasingly, pathologic and transcriptomic data in multimodal frameworks [40,42]. Baseline or early response imaging markers derived from AI and radiomic analysis have shown promise for predicting prognosis, treatment response, tumor phenotype, including tumor mutational burden, immune-related adverse events, and proxies of the immune microenvironment such as CD8 infiltration [43]. This range of endpoints, if validated prospectively, would substantially broaden the clinical utility of routine staging scans. For melanoma specifically, a realistic pathway to clinical adoption will likely require prospective multi-institutional datasets with standardized acquisition protocols, predefined segmentation workflows, and outcome endpoints determined before model training, consistent with the same standard of evidence demanded od any biomarker entering the clinical decision-making pathway.

## 4. Imaging for Detection of Primary Cutaneous Melanoma

The evaluation of primary cutaneous melanoma relies on a stepwise approach that combines clinical examination with adjunctive imaging techniques. The following techniques play an important role in enhancing diagnostic accuracy and guiding biopsy decisions (Table 1).

**Table 1:** Skin imaging for primary lesion detection.

Modality/Clinical Role/Level of Evidence (LOE)	Sensitivity/Specificity (95% CI), Detection Rate, or Concordance Rate	Preferred Stage Indication	Key Limitations and Contraindications	Adoption Status
**Clinical skin examination** *ABCDE criteria; ugly duckling sign* Whole-body visual inspection; first-line lesion recognition for all patients LOE: **High**	Sn 71% (59–82%)%; Sp 81% (48–95%) Meta-analysis data assessing skin examination from Vestergaard et al. [51].	All stages—initial triage	Operator-dependent; ABCDE framework achieves only moderate sensitivity and misses lesions < 6 mm, symmetric, or amelanotic [52,53,54,55,56]	**Accepted**
**Dermoscopy** *Epiluminescence microscopy; dermatoscopy* Lesion-level diagnostic triage; a standard adjunct to skin examination that reduces unnecessary biopsies LOE: **High**	Sn 90% (80–95%); Sp 90% (57–98%) Meta-analysis pooled diagnostic OR ≈ 15.6 years vs naked eye [51]	All stages—primary detection and biopsy triage	Requires structured training; limited imaging depth; benign nevi may be falsely diagnosed as melanoma by inexperienced users [51,55,57,58,59]	**Accepted**
**Total-body photography** *TBP; digital follow-up two-step method* Longitudinal monitoring in high-risk patients requires a photographic baseline for interval comparison LOE: **Moderate**	Sn > 90%; Sp 34.4% (32–36.9%) Positive biopsy rate ≈ 15% Moderate specify resulting in unnecessary biopsies; non-biopsied lesions excluded from most studies [60]	Stage 0–I with high-risk features (multiple atypical nevi or prior melanoma)	Requires specialized equipment and patient follow-up; inconsistent evidence on biopsy rate reduction; suboptimal for flexural or scalp lesions [60,61,62,63]	**Accepted (selective)**
**Reflectance confocal microscopy** *RCM; optical biopsy;* in vivo *microscopy* Second-level adjunct for dermoscopically ambiguous lesions; reduces unnecessary excision LOE: **Moderate**	Sn 92% (91–93%); Sp 70% (69–71%) Meta-analysis of 32 studies [64]	Stage 0–I with ambiguous dermoscopic lesions; lentigo melanoma evaluation	Specialized training required; limited availability outside tertiary centers; depth restricted to epidermis/superficial dermis; high cost [55,64,65,66,67]	**Accepted (selective)**
**Electrical impedance spectroscopy** *EIS; Nevisense device* Adjunctive biopsy decision support for equivocal lesions; FDA-approved (Nevisense) LOE: **Moderate**	Sn 96.6% (one-sided lower bound ≥ 94.2%); Sp 34.4% (32.0–36.9%) [68]	Stage 0–I with ambiguous lesions as an adjunct to dermoscopy	Not standard practice; limited adoption despite FDA clearance; moderate specificity increases unnecessary biopsies; high equipment cost [68,69,70]	**Accepted (selective)**
**Smartphone applications** *SkinVision, MoleMapper, UMSkinCheck* Consumer-facing self-monitoring tool; not validated for diagnostic use; educational adjunct only LOE: **Low**	Sn 80% (63% to 92%); Sp 78% (67% to 87%) [71]	Not stage-specific—patient self-monitoring only; not for clinical diagnosis	Unreliable diagnostic accuracy; high rate of non-evaluable images; false reassurance may delay presentation; not recommended for standalone diagnosis [71,72,73,74,75]	**Investigational**

Abbreviations: Sn = sensitivity; Sp = specificity; LOE = level of evidence. Adoption status: Accepted = recommended in current NCCN and/or ESMO guidelines for routine clinical use; Accepted (selective) = guideline-supported for defined high-risk subpopulations; Investigational = evidence base insufficient for routine clinical recommendation; use limited to research or specialist settings. Level of evidence (LOE): High = supported by meta-analyses and/or multiple prospective studies; Moderate = supported by prospective or large retrospective studies; Low = supported by small retrospective studies or systematic reviews with high heterogeneity.

### 4.1. Skin Examination

Considering the high cure rate of early-stage cutaneous melanoma with surgical excision [52], periodic skin examinations play an important role in early detection, often acting as the first step in identifying suspicious cutaneous lesions. Skin examinations typically consist of physicians inspecting a patient’s entire body, paying special attention to areas with abnormal lesions. Today’s methodology was introduced in 1985, when the ABCD criteria were implemented to standardize evaluations, specifically focusing on lesions that were asymmetrical, had border irregularities, color variations, and diameters greater than 6 mm. In 2004, this framework was expanded to become the ABCDE criteria, adding the evolution of lesions over time as a consideration [52,53,57,76]. Furthermore, the “ugly duckling sign” lesions—lesions that appear different from a patient’s typical moles—helps with the detection of melanomas that may not fit the ABCDE criteria [54]. Additionally, patients can be trained to perform self-skin examinations, improving the likelihood of early detection without routine physician evaluations.

While skin evaluations are easily accessible and inexpensive, they remain highly operator-dependent, with accuracy varying based on physician clinical experience. Reported sensitivities range from approximately 75% to 92%, while specificities range from 80% to 96%, depending on the clinical setting, reflecting a tradeoff between early detection and overdiagnosis [55]. Compared to other early detection tools, clinical examination alone is associated with lower specificity and may result in unnecessary biopsies of noncancerous nevi. Similarly, the ABCDE framework achieves only moderate sensitivity (approximately 65–80%) and may fail to identify melanomas smaller than 6mm, symmetrical, or uniformly pigmented [56]. While full-body skin examinations are essential for early detection, clinical examination alone is often insufficient for definitive diagnosis and is typically supplemented by dermoscopy and other imaging modalities to improve accuracy.

### 4.2. Dermoscopy

Dermoscopy, also known as dermatoscopy or epiluminescence microscopy, is a noninvasive imaging technique commonly used during initial full-body skin examinations. Dermoscopy enables visualization of skin structures beneath the outermost layer of the epidermis that are not visible to the naked eye. By reducing light reflection from the stratum corneum, dermoscopy enhances visualization of pigment networks, vascular patterns, and other morphological features within the epidermis, dermoepidermal junction, and superficial papillary dermis [57,76].

Dermoscopy has been shown to improve the diagnostic accuracy of both melanocytic and nonmelanocytic cutaneous lesions compared to naked-eye exams. Metadata analyses have demonstrated a significantly greater diagnostic odds ratio for melanoma (up to 15.6), and clinical studies have shown improved sensitivity (82.6% vs. 70.5%) and overall diagnostic accuracy (AUC 0.89 vs. 0.83) when dermoscopy is combined with standard visual examinations [51,58]. Pattern recognition algorithms, including the seven-point checklist, which assesses features such as changes in size, shape, and color along with additional clinical signs, have been created to standardize dermoscopic interpretation and improve diagnostic consistency among clinicians [55]. Similarly to skin examination, dermoscopy is heavily dependent on a provider’s training and experience. Users with limited experience are subject to misinterpreting dermoscopic findings. For example, benign pigmented lesions may be mistaken for malignant lesions, resulting in unnecessary biopsies or, conversely, malignant features such as irregular streaks, atypical vascular structures, and a blue-white veil may be overlooked, resulting in missed lesions that warrant biopsy [59]. Despite this limitation, dermoscopy plays an important role in the early diagnosis of cutaneous lesions, serving as the primary adjunct to routine skin examination.

### 4.3. Total-Body Photography

Total-body photography (TBP) is a commonly used screening modality that aims to help with the detection of melanocytic lesions in high-risk patients, such as those with a history of prior melanoma, multiple or atypical nevi, fair skin, increased ultraviolet exposure or genetic risk factors. The International Dermoscopy Society recommends the use of digital monitoring with TBP and sequential dermoscopic imaging in patients with ≥60 nevi or a CDKN2A mutation, and in selected patients with >40 nevi who possess additional melanoma risk factors [77]. TBP involves standardized, full-body imaging to establish a baseline set of images, which can be compared with future TBP or full-body skin exams. This longitudinal comparison is especially useful for patients with numerous nevi, enabling the identification of new lesions and changes in existing lesions over time [60,61].

Advantages of TBP include improved detection of early-stage thin melanomas, as well as better overall survival, with some studies suggesting a reduction in unnecessary biopsies [60,61]. In a systematic review including 10 studies and over 41,000 patients, TBP demonstrated a mean positive biopsy rate of approximately 15%, reflecting relatively high specificity in high-risk patients [60]. However, measurements of diagnostic accuracy, including sensitivity and specificity, remain difficult to determine, as most studies do not include data on non-biopsied lesions. As a result, TBP is primarily considered an adjunctive surveillance tool, with its greatest benefits derived from its ability to enable longitudinal monitoring and enhance diagnostic precision through objective visual comparisons.

The limitations of TBP include the need for specialized equipment, time investment, and consistent patient follow-up. TBP may also be less effective at capturing the progression of cutaneous lesions in certain anatomical regions. Additionally, evidence regarding its impact on biopsy rates is conflicting, with some studies showing no reduction compared to standard care [60,62,63].

### 4.4. Electrical Impedance Spectroscopy

Electrical impedance spectroscopy (EIS) is an emerging, noninvasive technique that evaluates tissue architecture by measuring differences in the electrical properties of skin lesions, reflecting underlying structural and cellular changes. Considering that malignant tissue exhibits altered electrical resistance and capacitance due to variations in cellular structure, EIS has been proposed as a tool to determine whether biopsied lesions are suspicious for melanoma [69,70]. EIS devices, such as the commercially available, FDA-approved Nevisense, have shown a high level of sensitivity in detecting melanoma, often exceeding 90%, but low specificity (34.4%; 95% CI, 32–36.9%). Specificity remains moderate, contributing to false-positive results, resulting in unnecessary biopsies. [68] Given that EIS is costly and has limited availability, it is not widely used as a standalone diagnostic tool but is considered as an emerging supplementary tool that could guide biopsy decisions [70].

### 4.5. Reflectance Confocal Microscopy

Reflectance confocal microscopy (RCM) is a noninvasive imaging technique that uses confocal laser scanning at various wavelengths to provide real-time visualization of the epidermis and superficial dermis at near-cellular resolution. While originally developed for high-resolution imaging of tissues, early applications of RCM demonstrated that the modality could create “optical biopsies” of human skin, with melanin serving as a strong natural contrast agent due to its high refractive index [65]. Once introduced clinically, RCM was first used as a tool to supplement dermoscopy, specifically for lesions with unclear clinical or dermoscopic features, where it improved diagnostic accuracy by identifying cellular features not visible with dermoscopy and reduced unnecessary excisions [66]. In the past decade, growing evidence has supported the use of RCM as a highly accurate noninvasive diagnostic adjunct, leading to its expanded role in the evaluation of clinically and dermoscopically equivocal skin lesions [64,66,67,78].

RCM has shown high sensitivity and moderate-to-high specificity for melanoma detection. In a meta-analysis including 32 studies, Pezzini et al. noted the pooled sensitivity and specificity to be approximately 92% (95% CI: 0.91–0.93) and 70% (95% CI: 0.69–0.71), respectively [64]. Similarly, Borsari et al. demonstrated the sensitivity and specificity of RCM in a prospective study evaluating melanoma detection to be 95.3% and 83.9%, respectively [66]. These findings suggest that RCM not only improves diagnostic accuracy but may also reduce the number of unnecessary excisions compared to dermoscopy alone.

Despite the diagnostic benefits of RCM, the technique has several important limitations. First, accuracy depends on the operator and specialized training. RCM is not used on a daily basis in most dermatology clinics because its use is generally reserved for specific cases. Consequently, dermatologists have limited training and experience in accurately acquiring and interpreting RCM images. Second, RCM is often expensive and has limited availability compared to other noninvasive diagnostic tools such as dermoscopy. Lastly, RCM’s limited depth of penetration allows evaluation of only the epidermis and superficial dermis, preventing assessment of deeper tumor invasion. Nonetheless, RCM remains a valuable diagnostic tool whose clinical utility could be enhanced through future research aimed at improving accessibility, standardization across studies, and integration with AI.

### 4.6. Cell Phone Apps

Smartphone applications have emerged as cheap, easily accessible resources for melanoma risk assessment, using smartphone cameras and, in some cases, AI to help patients with early diagnosis and surveillance of malignant melanoma. Commonly used phone applications include SkinVision, Miiskin, MoleMapper, and UMSkinCheck [72,73,74]. These mobile apps enable patients to take pictures of skin lesions, track changes over time, and receive either an algorithmic-based risk assessment or an evaluation from a dermatologist through teledermatology [72,73].

While their accessibility and potential to increase patient engagement in early melanoma detection make these novel tools attractive and promising, current evidence does not support the use of smartphone applications as standalone diagnostic tools for melanoma. In a systematic review including nine studies that evaluated over 700 lesions across six smartphone apps, Freeman et al. reported that these applications demonstrated variable and generally limited diagnostic accuracy because the studies had many limitations, such as small sample sizes, poor study design, and high rates of unevaluable images [71]. Although some applications have demonstrated moderate sensitivity, overall diagnostic performance remains inconsistent, with studies showing that nearly 30% of melanomas are classified as low risk, raising concerns regarding false reassurance that may delay care and false-positive findings that inflict unnecessary anxiety on patients [71,75]. Consequently, cell phone applications should not be used for definitive diagnosis but may benefit patient care by acting as educational resources that facilitate self-monitoring, particularly in settings with limited access to dermatologic care.

## 5. Imaging Techniques for Detection of Metastasis

While the aforementioned imaging techniques are relevant for staging the initial primary melanoma, other imaging techniques are more useful for the detection of metastasis or recurrence. These techniques are described below and summarized in Table 2.

**Table 2:** Imaging for staging and metastasis detection.

Modality/Clinical Role/Level of Evidence (LOE)	Sensitivity/Specificity (95% CI), Detection Rate, or Concordance Rate	Preferred Stage Indication	Key Limitations and Contraindications	Adoption Status
**Staging and metastasis detection**				
**Chest X-ray** *CXR; chest radiograph* Formerly used for the detection of lung metastases; replaced by CT in current guidelines LOE: **Moderate**	Sn 50% (7–93%); Sp 96% (95% CI: 94–98%) [79].	Not routinely recommended at any stage per current NCCN/ESMO guidelines	Poor sensitivity for pulmonary metastases; does not alter management; not cost-effective when combined with physical exam; harmful ionizing radiation exposure [79,80,81,82,83,84,85]	**Inconsistently used**
**Computed tomography** *CT; whole-body CT* Cross-sectional systemic staging; surveillance imaging in high-risk patients; standard for thoracic/abdominal disease LOE: **High**	Sn Staging (LN): 9% (1–52%); Surveillance (LN): 61% (15–93%); Sp Staging (LN): 92% (50–99%); Surveillance (LN): 97% (70–100%) from [11].	Stage IIB–IV—systemic staging and surveillance	Low biologic specificity; cannot differentiate inflammatory from malignant lesions; cumulative ionizing radiation during long-term surveillance; iodinated contrast allergy/renal impairment risk [11,55,86,87,88,89]	**Accepted**
**Magnetic resonance imaging** *MRI; whole-body MRI; brain MRI* Preferred modality for intracranial metastasis detection; soft tissue and bone marrow evaluation; recommended for CNS surveillance LOE: **High**	Only point estimates available: MRI sensitivity 73.4% vs. CT sensitivity 78.2% (*p* = 0.0744), and MRI specificity 83.4% vs. CT specificity 50.4% (*p* < 0.0001) [90].	Stage IIC–IV (brain imaging); stage III–IV (soft tissue/bone marrow); annual brain MRI recommended for stage IIB–IV (NCCN)	Long scheduling lead time (≈20 vs. ≈5 days for CT); high cost; contraindicated with certain implants/pacemakers; gadolinium retention concerns [16,90,91,92,93,94]	**Accepted**
**Ultrasound** *Regional nodal US; high-frequency US; US ± FNAC* Regional nodal assessment and real-time biopsy guidance; pre-operative Breslow estimation in selected cases LOE: **High**	US alone: Sn 35.4% (17.0–59.4%); Sp 93.9% (86.1–97.5%). US + FNAC: Sn 18.0% (3.58–56.5%); Sp 99.8% (99.1–99.9%). Note: Adding FNAC substantially reduces sensitivity while improving specificity [78].	All stages with palpable or suspicious nodes; complementary to cross-sectional imaging	Not a whole-body staging tool; operator-dependent; cannot detect micrometastatic disease; bowel gas limits abdominal assessment [55,90,95,96,97,98,99]	**Accepted**
**FDG PET/CT** *[18F]-fluorodeoxyglucose PET/CT* Functional whole-body staging; surveillance in advanced disease; treatment response assessment adjunct; not a substitute for SLNB LOE: **High**	Sn Overall: 81% (73–87%). Distant mets: 88% (81–93%). Regional LN: 56% (40–72%). Sp Overall: 92% (90–94%). Distant mets: 94% (91–96%). Regional LN: 97% (94–99%) [17].	Stage III–IV—systemic staging, surveillance, and treatment response; not indicated for micrometastatic nodal disease (PET cannot replace SLNB)	Cannot detect micrometastatic nodal disease; imaging limited to about 5 mm lesions; false positives with inflammatory lesions (especially on immunotherapy); ionizing radiation; contrast allergy; not cost-effective in low-risk populations [11,17,18,100,101,102,103,104,105]	**Accepted**
**Lymphoscintigraphy** *99mTc-nanocolloid scintigraphy; planar lymphoscintigraphy* Pre-operative sentinel lymph node localization: an essential component of the SLNB surgical workflow LOE: **High**	SPECT/CT DR: 98.28% (95% CI, 97.94–99.19%) vs. planar lymphoscintigraphy DR: 95.53% (95% CI, 92.55–97.77%)[106]. No stats for Sn or Sp as lymphoscintigraphy is not a standalone technique and is combined with SLNB.	T1b to T4 stage—pre-operative SLN mapping; required before SLNB in most protocols	Procedure-specific (not for systemic staging); requires nuclear medicine facility; ionizing radiation; limited anatomic resolution in complex regions [107,108,109,110,111]	**Accepted**
**SPECT/CT** *Single-photon emission CT/CT hybrid; tomographic lymphoscintigraphy* Improved 3D SLN localization in anatomically complex regions; EANM-recommended for groin and axillary mapping LOE: **Moderate**	SPECT/CT DR: 98.28% (95% CI, 97.94–99.19%) vs. planar lymphoscintigraphy DR: 95.53% (95% CI, 92.55–97.77%) [106].	Stage IB–III. —SLN mapping in complex anatomy (head/neck, axilla, and groin)	Added time and cost; limited availability outside specialist centers; conflicting data on disease-free survival benefit; not for systemic staging [19,106,112,113,114,115,116,117]	**Accepted**

Abbreviations: Sn = sensitivity; Sp = specificity; SLNB = sentinel lymph node biopsy; SLN = sentinel lymph node; FNAC = fine-needle aspiration cytology; EANM = European Association of Nuclear Medicine; LOE = level of evidence; FDG = fluorodeoxyglucose; PET = positron emission tomography; CT = computed tomography; MRI = magnetic resonance imaging; SPECT = single-photon emission computed tomography; NCCN = National Comprehensive Cancer Network; ESMO = European Society for Medical Oncology. Adoption status: Accepted = recommended in current NCCN and/or ESMO guidelines for routine clinical use; Inconsistently used = used but not recommended in current guidelines. Level of evidence (LOE): High = supported by meta-analyses or multiple prospective studies; Moderate = supported by prospective or large retrospective studies.

### 5.1. Chest X-Rays

Chest X-rays (CXRs) are photographs taken using X-rays, an ionizing radiation with a wavelength of 0.01–10 nm. X-rays are typically generated by heating a tungsten filament to 2,200°C to produce high-energy electrons that move through an applied voltage towards an anode, releasing X-ray energy upon collision that penetrates the imaged body area [80,81,118]. X-ray images provide more contrast than typical photographs and allow visualization of an individual’s internal organs, making them a useful imaging technique for diagnostic purposes. CXRs were historically conducted frequently in clinics to stage patients and detect lung metastases in patients with high-risk melanoma but are no longer commonly used due to their poor performance [81].

Several studies have found that CXRs do not accurately stage melanoma patients or identify metastases [81]. One study showed that CXRs had a sensitivity and detection rate of only 50%, [95% CI, 7%, 93%] in staging cutaneous melanoma patients [79,82]. Another study showed that pre-operative CXR was not only unable to identify pulmonary metastases but also did not change treatment strategies in patients treated for primary melanoma [83]. During follow-up, CXRs detected less than 10% (3.2–7.4%) of metastases [84].

Despite the limited accuracy of CXRs, one advantage of this imaging technique is that CXRs are relatively cheap compared to other techniques for both initial staging and follow-up [81,84]. However, one study demonstrated that combining physical examination with CXR is not cost-effective [85], suggesting that CXR may be cheap but not useful enough to justify the cost. Further, CXRs have high false-positive rates, which could cause excessive anxiety for patients [79,82]. Overall, it appears that CXR is not very beneficial for staging; it is no wonder that CXR has been largely removed from standard clinical practice.

### 5.2. Computed Tomography

CT allows anatomical visualization of the interior of the human body by combining multiple X-ray images taken from a rotating X-ray scanner that generates and detects X-ray passing through every angle of the patient’s body. Image reconstruction techniques transform data on detected photon intensity into pixel brightness values linearly to produce clear, high-contrast photographs [86,119,120].

CT has low specificity, correctly staging only 24% [95% CI, 19% to 30%] of patients with palpable, proven lymph node metastases of melanoma, and a moderate sensitivity of 78%, [95% CI, 69%, 87%] [55,87]. The usefulness of CT in melanoma clinical practice remains controversial. One study showed that CT alone could not be used to diagnose SLN metastases; however, other studies have concluded that CT has a high specificity of 93% (higher than FDG PET) and is suitable for metastasis detection in melanoma patients [87,88,89]. Another study showed that using CT in combination with another imaging technique, e.g., FDG PET/CT, results in higher accuracy for metastasis detection compared to CT alone in patients at high risk of developing melanoma [55]. More research needs to be conducted on CT-based metastasis detection accuracy to see if a clear consensus in the literature will emerge that can more definitively inform any changes in clinical practice guidelines.

Despite its debatable accuracy, its ease of use and clear, interpretable images make CT a current standard imaging technique. CT is cost-effective: adding CT to the diagnostic workup was shown in one study to decrease costs by 5.5% compared to when no diagnostic tests were used [121].

### 5.3. Magnetic Resonance Imaging

MRI involves subjecting the patient to a strong external magnetic field (created by the MRI scanner), which aligns the axes of ^1^H nuclei either parallel or perpendicular to the magnetic field. A radio wave is applied perpendicularly to the external magnetic field to deflect the magnetic vector, causing some hydrogen nuclei to resonate and transition into a higher-energy state. Turning the radio wave off allows the hydrogen nuclei to return to their original positions, emitting energy as a voltage that can be detected by a circular wire coil surrounding the patient and converted using mathematical algorithms into a reconstructed image [91,122].

Overall, MRI is relatively accurate in detecting metastases in melanoma patients. While MRI is less sensitive than CT, it is more specific [90]. It has been shown that MRI detects more metastatic sites than CT in the liver, spleen, subcutaneous tissue, muscle, bone marrow, and brain and impacts treatment decisions. MRI is also the recommended imaging modality for detecting brain metastases [16,92]. The relatively high performance of MRI makes it a suitable technique for melanoma staging and should remain a part of clinical guidelines.

However, one downside is that MRI scans tend to take longer to schedule than other imaging techniques. On average, it takes 20 days between when a patient books an appointment and the scheduled date for an MRI scan, compared to 5 days for CT [93]. Another study analyzing the costs of various procedures for melanoma patients found that brain MRI was one of the most expensive procedures ($4066.00), along with FDG PET ($5121.40) [123]. Although MRI is generally more cost-effective and indicated in later stages, especially stage III, it is still done for early-stage melanoma patients with concerning symptoms. The increased accuracy of MRI should be weighed against the increased cost, as it can influence treatment for some patients [94].

### 5.4. Ultrasound

Ultrasound (US) involves emitting pulses of sound (typically 1–15 MHz) from a transducer, which will penetrate the patient’s tissue until they reach an interface that propagates back a proportion of the sound energy as an echo pressure wave with a particular voltage that can be detected [95]. This allows calculation of the depth of the interface, and these calculations from many pulses and echoes can be combined into a single image, where brightness represents the depth of the reflecting surface in the body and is proportional to the detected voltage.

Several studies have suggested that US is useful in assessing primary melanoma prior wide excision. US has relatively high accuracy in assessing the depth of thicker tumors (Breslow depth > 0.75 mm) [96] and can detect LN invasion with greater accuracy and discriminatory power than clinical exam [97]. Combining US with fine-needle aspiration cytology (FNAC) before SLNB had a specificity of 99.8%, [95% CI, 99.1%, 99.9%] (but a sensitivity of only 18.0%, [95% CI, 3.58%, 56.5%]), while US alone had a specificity of 93.9%, [95% CI, 86.1%, 97.5%] and a sensitivity of 35.4%, [95% CI, 17.0%, 59.4%] detecting nodal disease. This suggests that US might be better used in combination with other techniques rather than alone in patients with cutaneous melanoma [124].

Compared to a number of imaging techniques, US is relatively cheap [98]. A study by Hengge et al. showed that LN US is cost-effective at all stages and that abdominal US is not cost-effective for early-stage patients. Additionally, they showed that reducing CXRs, abdominal US, and blood tests in the early stages saved more than $120,000 annually [99].

### 5.5. Fluorodeoxyglucose Positron Emission Tomography

FDG PET is a type of PET scan that uses FDG as a radiotracer to measure increased glucose uptake based on the detected radioactive decay of FDG circulating in the vascular system after intravenous administration [100]. Glucose uptake is typically increased in inflamed and cancerous cells due to high glycolytic activity, making FDG a highly useful and common radiotracer for diagnosis [100,101].

FDG PET is quite accurate in detecting lesions, regional disease, and distant metastases. Fuster et al. showed that, using FDG PET, the sensitivity and specificity for lesion detection was 74% and 86%, respectively; the accuracy for detecting regional disease was 91%, and that for distant metastases was 85% [102]. For most regions in the body, FDG PET metastasis detection rates were significantly higher than those of individual current standard diagnostic procedures, with the exception of the lung (FDG PET had a sensitivity of 57%, compared to CT’s sensitivity of 93%). However, another study showed that combining FDG PET with CT resulted in an overall sensitivity of 87% and specificity of 82%, and a different study measured high sensitivity but low specificity for FDG PET compared with CT [87,103]. This suggests that FDG PET/CT may be a more useful imaging technique for lung lesion cases than CT or FDG PET alone, where the disadvantages of one imaging technique may be offset by the advantages of the other technique [87,102,103]. FDG PET use was also shown to influence treatment decisions for recurrent melanoma, suggesting that it has clinical value [102]. However, FDG PET was not shown to accurately evaluate regional metastases because it does not have the necessary microscopic resolution, but it is still useful for detecting distant metastases and therefore should still be used in the clinical management of melanoma patients [104]. FDG PET is often done in conjunction with CT; using FDG PET/CT resulted in a net saving of €1048 and avoided 20% of futile surgeries, indicating the usefulness and cost-effectiveness of incorporating FDG PET/CT into the routine clinical management of melanoma patients at high risk of metastasis [105]. While FDG PET has some limitations in its metastasis detection capacity, its benefits are compelling and justify its clinical usage.

### 5.6. Lymphoscintigraphy

Lymphoscintigraphy is an imaging technique in which nanocolloid technetium (^99m^Tc)-labeled radiotracers are injected subcutaneously or intradermally around the primary tumor site to visualize lymphatic flow and lymph nodes [107,108]. The radiotracer emits gamma rays that can be captured by a gamma camera to develop images and, during surgery, can be identified with a gamma probe [108].

Several studies have found lymphoscintigraphy to be an accurate method of localizing LNs, including the sentinel lymph node (SLN), for which the localization accuracy is 98% [109,110,111]. One study found that when lymphoscintigraphy was conducted twice in melanoma patients, the findings were similar in 96%, [95% CI, 91–100%] of study participants, suggesting a high level of consistency with this imaging technique [111].

Lymphoscintigraphy has several advantages over other LN imaging techniques: lymphoscintigraphy radiotracers rarely cause allergic reactions, never cause pulmonary embolisms and the procedure is safe for individuals with pulmonary dysfunctions [108]. A study by Stoffels et al. showed that the median cost of lymphoscintigraphy combined with SLN excision was €2330.2, with a cost saving of 30% when SPECT/CT was added (€1619.7), suggesting that lymphoscintigraphy might be more cost-effective when used in conjunction with other imaging techniques. The cost difference can be explained by differences in hospital stay and surgical procedure costs that offset the increased cost of pre-operative imaging [112]. Despite this cost disadvantage, the high accuracy of lymphoscintigraphy and its unique safety advantages make it a highly valuable imaging technique for melanoma care.

### 5.7. SPECT/CT

SPECT/CT imaging is a hybrid of tomographic lymphoscintigrams (SPECT) and CT data. It can be used to localize SLNs [113]. Radiotracers are injected into the skin, emitting gamma rays that can be detected to create 3D photographs. These 3D photographs are then combined with data from a traditional CT scan, which provides much finer anatomical resolution and creates a robust and accurate imaging technique [114].

SPECT/CT has been shown to be even more accurate than lymphoscintigraphy in staging melanomas. It detects significantly more SLNs and, when used in conjunction with SLNB and lymphoscintigraphy, improves disease-free survival and decreases relapse rates compared to SLNB performed using lymphoscintigraphy alone [19,106,115,116]. Conversely, a study by Chapman et al. found no difference between SPECT/CT and lymphoscintigraphy in melanoma recurrence rates or disease-free survival [116]. This discrepancy is likely explained by the difference in follow-up period length; Chapman et al. used an average follow-up period of only 10 months in their SPECT/CT group, compared with 48 months in the primary study by Stoffels et al., which suggested that SPECT/CT improves disease-free survival [19,106,115,116]. Other differences between these two studies include the melanomas studied (head and neck melanomas in Chapman et al. compared with head, neck, torso, hand, foot, and extremity melanomas in Stoffels et al.) and sample size (n = 85 in Chapman et al. compared with n = 403 in Stoffels et al.). Overall, this comparative analysis indicates that SPECT/CT demonstrates long-term but not short-term advantages in disease-free survival compared to lymphoscintigraphy. Further, this advantage may depend on the localization of the melanoma, as head and neck melanomas seem less likely to benefit from SPECT/CT.

One downside is that SPECT/CT is time-consuming and more costly than other imaging techniques. However, its increased accuracy may prove to be cost-effective when used for difficult or unusual cases; thus, its clinical usage is rarer but still indicated for certain cases as needed [87,117].

### 5.8. Photoacoustic Imaging

Photoacoustic imaging (PAI) is a relatively novel noninvasive imaging technique that utilizes the absorption of nano-pulsed excitation light by chromophores or exogenous contrast agents, resulting in a transient increase in temperature and tissue expansion. These changes generate photoacoustic signals that may be measured by US, enabling the development of two- and three-dimensional images unique to the optical properties of the tissue being evaluated [125].

Due to its ability to provide detailed imaging of the microvasculature, tumor boundaries, tissue architecture, and circulating tumor cells, PAI has emerged as an adjunctive imaging tool for the evaluation of cutaneous melanoma. In particular, it has demonstrated utility in providing valuable information on primary tumor depth of invasion that is closely correlated with histopathologic Breslow thickness [125,126,127]. However, while PAI can visualize primary melanoma lesions and the surrounding vasculature, current evidence supporting its standalone diagnostic accuracy for melanoma compared with nonmalignant lesions remains limited [127].

In contrast, stronger clinical evidence exists for the use of PAI in detecting metastatic disease, particularly in SLNs. Stoffels et al. reported that multispectral optoacoustic tomography (MSOT) achieved sensitivity approaching 100% for identifying metastatic involvement in SLNs, although specificity was lower, ranging from approximately 48% to 62%, reflecting an increased rate of false-positive findings [127,128]. Additionally, MSOT has demonstrated high concordance with conventional lymphoscintigraphy for SLN detection, supporting its potential as a nonradioactive alternative for nodal staging in melanoma patients [128].

Overall, PAI represents a promising noninvasive imaging modality with particular strength in assessing tumor depth and detecting metastatic involvement. However, its role in the primary diagnosis of cutaneous melanoma remains under investigation, and further large-scale, standardized studies are needed to better define its sensitivity, specificity, and overall clinical utility for the detection of primary cutaneous lesions.

### 5.9. Practical Clinical Applications

The aforementioned imaging techniques can be applied at various stages of the melanoma clinical workflow. These applications can be classified as pre-, intra-, or post-operative.

Pre-operative imaging techniques include SPECT/CT and lymphoscintigraphy. Historically, CXR was typically used to detect lung metastases in early-stage melanoma patients but is not recommended in current guidelines. Additionally, there has been some controversy surrounding routine CXR use in staging procedures, with evidence showing high false-positive rates and no significant improvement in survival rates among melanoma patients with pulmonary metastases [129,130]. While CXRs have occasionally been used post-operatively for surveillance imaging, several studies suggest that they have relatively low performance and provide minimal benefit for patients; thus, they should not be recommended in standard clinical guidelines [16,131,132]. On the contrary, SPECT/CT has been shown to be useful in pre-operative SLN mapping for primary cutaneous melanoma and is strongly recommended by the European Association of Nuclear Medicine (EANM) practice guidelines for groin and axillary imaging [19,133]. Lymphoscintigraphy is almost always used for pre-operative SLN basin identification. LNs are resected based on the “10% rule”, which states that all LNs with radioactivity at least 10% that of the most radioactive node should be removed [134].

Intra-operative imaging is rarer, but a recent study suggests that US performed during surgery can help identify excision boundaries and confirm complete tumor resection [135]. Without intra-operative US, it is possible that some tumor tissue remains undetected, leading to incomplete resection that must be corrected by a second surgery, particularly for cases where pre-operative imaging results in ambiguous, insufficient localization of the tumor or nodal metastases. Intra-operative US might be more cost-effective and efficient than standard surgical procedures that do not include intra-operative imaging, as it can reduce the likelihood of repeat surgery in some patients. US is primarily used post-operatively for early detection of melanoma recurrence and has also been used pre-operatively for accurate Breslow thickness estimation [136,137].

CT, FDG PET/CT, and MRI are all used both pre- and post-operatively in melanoma care. Pre-operatively, CT and FDG PET/CT provide high-resolution anatomical visualization that is useful for the baseline evaluation of stage III-IV melanomas based on the AJCC staging system [16]. MRI examination is also recommended for stage IIC or higher-risk melanoma. For post-operative and follow-up imaging, whole-body CT and FDG PET are indicated for the detection of metastases. Brain MRI is also indicated specifically for the detection brain metastases; the NCCN guidelines recommend yearly brain MRI for the first five years in patients with stage IIB-IV melanoma [16,138].

### 5.10. Comparison of Metastasis Detection Imaging Techniques

Overall, no single imaging modality is optimal for all aspects of melanoma metastasis detection, and each technique demonstrates distinct strengths and limitations. Though CXR has been used historically, its performance is significantly lower than that of other imaging modalities, and it has generally been replaced by CT, which has high specificity while being cost-effective.

Although CT is highly accurate, several other imaging techniques are useful for detecting metastases in areas where CT shows limited usability. MRI allows better identification of metastasis than CT in multiple organs, such as the liver or brain. Similarly, lymphoscintigraphy should be used for the detection of LN metastases specifically. Alternatively, SPECT/CT can also be used for LN metastasis detection and may, in fact, perform even better than lymphoscintigraphy over the long term.

FDG PET and FDG PET/CT are used for similar reasons to MRI. Like MRI, the use of both techniques is decided based on the suspected metastatic site. FDG PET is most suitable for evaluating distant (rather than regional) metastases to complement CT imaging, while FDG PET/CT is most useful for identifying suspected lung lesion cases. However, unlike MRI, FDG PET and FDG PET/CT provide the advantage of functional imaging to visualize metabolic activity in addition to (lower-resolution) anatomic structure.

US is an interesting case. Although its specificity is exceptionally high, even when used alone, and increases further when combined with FNAC, its sensitivity is lower than that of MRI or CT. Given its high specificity, US might be better suited for “rule-in” diagnostic testing compared to MRI or CT. This also corroborates its use in advanced stages compared with CT, which can be used in earlier stages.

PAI is a recent imaging technique that remains at the investigational stage, and its clinical applications have yet to be definitively established. Thus, it is not yet clear where in the clinical workflow PAI will be most beneficial.

## 6. Future Applications

New advancements have occurred in recent years to improve melanoma diagnosis and staging. These techniques are described below, with a summary of the important features in this section detailed in Table 3.

**Table 3:** Future/experimental applications for tumor assessment.

Modality/Clinical Role/Level of Evidence (LOE)	Sensitivity/Specificity 95% CI, Detection Rate, or Concordance Rate	Preferred Stage Indication	Key Limitations and Contraindications	Adoption Status
**Photoacoustic imaging** *PAI; multispectral optoacoustic tomography (MSOT)* Tumor depth and vascularity assessment; SLN detection as a potential nonradioactive alternative to lymphoscintigraphy LOE: **Very low**	Concordance rate of ICG-MSOT vs. Tc-99m was 94.6% at the basin level and 96.4% at the node level [128]; limited large-scale validation; high false-positive rate for SLN is a current barrier	Research/specialist centers—stage I–III (tumor depth estimation, SLN mapping)	Not commercially available; standalone diagnostic accuracy for primary melanoma not established; requires further prospective evaluation [125,126,127,128]	**Investigational**
**Non-FDG PET** *[^18^F]-FDOPA, [^18^F]-FCH, [^11^C]-choline PET* Melanin-targeted or receptor-specific functional imaging; potentially superior for FDG-avid-negative melanoma subsets LOE: **Very low**	Comparable or superior to FDG PET in selected case series Primarily case reports and small series; no prospective study conducted in melanoma populations	Selected advanced-stage patients—FDG-avid-negative or metabolically atypical disease	Short radiotracer half-life (11C ≈ 20 min requires on-site cyclotron); high cost; limited availability; no large-cohort validation in melanoma [101,139,140,141,142,143,144]	**Investigational**
**AI-assisted imaging** *Deep learning; CAD; dermoscopy AI* (e.g., *DermaSensor)* Dermoscopic decision support; diagnostic triage augmentation; one FDA-approved device [145] LOE: **Low**	Derm-CAD (dermoscopy-based, 22 studies): Sn 90.1% (95% CI, 84.0–94.0%); Sp 74.3% (95% CI, 63.6–82.7%) MSI-CAD (multispectral imaging, eight studies): Sn 92.9% (95% CI, 83.7–97.1%); Sp 43.6% (95% CI, 24.8–64.5%) [146]	All stages—decision-support adjunct only; not approved for standalone diagnosis	Limited by dataset bias; no external validation; overfitting risk; no cross-platform standardization; medico-legal uncertainty; explainability concerns; patient trust barriers; additional cost vs. standard care [145,146,147,148,149,150,151,152,153,154,155,156,157,158,159]	**Investigational**
**Image-guided surgery** *NIR fluorescence (ICG-based); intra-operative US; radio-guided surgery; 5-ALA fluorescence* Intra-operative tumor margin delineation; real-time SLN localization; confirmation of complete resection; reduction in re-excision rates LOE: **Low**	NIR fluorescence SLN detection: concordance with lymphoscintigraphy in 97.1% of nodes [160] US-guided biopsy: greater mean resected tumor thickness vs. non-guided (2.9 mm vs. 2.1 mm) [161]; intra-operative US margin confirmation reduces incomplete resection [135] Fluorescence-guided resection: SLN identification success rate 100% in NIR swine model [110]; recurrence-free outcomes demonstrated in murine IGS vs. non-IGS cohorts [162,163]	Stage IB–III Intra-operative SLN localization and margin assessment Stage III–IV real-time resection guidance for metastases removal; any stage requiring re-excision risk reduction	Not a standalone staging modality; technique-specific learning curve; ICG requires fluorescence-capable surgical equipment not universally available; 5-ALA is limited to specialist centers; radio-guided SLNB requires nuclear medicine co-ordination; most high-quality evidence comes from murine or small prospective single-center series; no RCT directly comparing IGS to standard resection for OS or DFS [135,160,161,162,163,164,165,166]	**Investigational**
**Radiomics** *High-throughput quantitative feature extraction (texture, shape, intensity heterogeneity) from CT, MRI, and FDG PET/CT* Applied to treatment response prediction, prognostic stratification, and pseudoprogression discrimination LOE: **Low**	Pooled AUC 0.83 (95% CI: 0.74–0.92) across 24 melanoma studies for treatment response and survival prediction [40]	Stage III–IV; systemic therapy response prediction and prognostic modeling in the advanced disease setting	No validated feature extraction standards across scanners or reconstruction protocols despite IBSI harmonization efforts [49,50]	**Investigational**

Abbreviations: Sn = sensitivity; Sp = specificity; SLNB = sentinel lymph node biopsy; SLN = sentinel lymph node; FNAC = fine-needle aspiration cytology; MSOT = multispectral optoacoustic tomography; CAD = computer-assisted diagnosis; AI = artificial intelligence; RCT = randomized controlled trial; LOE = level of evidence; FDG = fluorodeoxyglucose; PET = positron emission tomography; CT = computed tomography; MRI = magnetic resonance imaging; SPECT = single-photon emission computed tomography; PAI = photoacoustic imaging. Adoption status: Investigational = evidence base insufficient for routine clinical recommendation; use limited to research or specialist settings. Level of evidence (LOE): Low = supported by small retrospective studies or systematic reviews with high heterogeneity; Very low = case reports, animal data, or single-center feasibility studies only.

### 6.1. Newer Modalities in Imaging: Non-FDG PET

PET imaging done without an FDG radiotracer is referred to as non-FDG PET. Some examples include [^18^F]-fluorocholine (FCH) PET and [^11^C]-choline PET. FDG is one of the most common radiotracers for PET imaging because it allows tracking of glucose uptake, which is typically upregulated in cancer cells. While FDG is very useful for most cancer imaging, it does have its limitations. First, FDG PET focuses on glucose uptake by cells in a manner that is not cancer-specific and can also occur in processes like inflammation. Non-FDG PET circumvents this issue, as it does not use a glucose radiotracer but rather [^15^O]-water, [^13^N]-ammonia, and other radioactive isotope-labeled compounds [101]. Second, some tumors do not have significant FDG uptake and may therefore be detected by non-FDG PET [101,139,140,141]. This is particularly true for melanoma—non FDG-avid findings are more likely to occur in melanomas compared to other types of cancers [142]. For instance, FCH PET has high levels of uptake in melanoma patients [101]. In addition, metastatic melanoma is less likely to be FDG-avid than non-metastatic melanoma [141].

The accuracy of non-FDG PET is comparable to, and in some cases better than, that of FDG PET. [^11^C]-choline PET has been shown to detect multiple myeloma at the same rate as FDG PET and to detect malignant sinonasal melanoma better than FDG PET in one case study, while [^18^F]-FDOPA PET has significantly higher accuracy than FDG PET in diagnosing primary brain tumors (e.g., glioblastoma) as well as metastatic brain tumors [101,139,140,143]. MET PET has a sensitivity of 100% for detecting lesions greater than 1.5 cm in diameter and 81% for detecting lesions of any size [101,144].

Some non-FDG PET types present practical challenges. For instance, [^11^C]-choline PET has a very short half-life of approximately 20 min, which means that it requires its own on-site cyclotron (which is not present at every PET center) [139]. This makes certain non-FDG PET scans impractical and costly to implement. Thus, the advantages of improved accuracy and the disadvantages of poor practicality and high financial costs of non-FDG PET should be taken into consideration when deciding the appropriate PET radiotracer for patients.

### 6.2. Image-Guided Surgery

Image-guided surgery (IGS) is a type of surgical procedure that uses imaging techniques simultaneously to guide surgery. Image guidance is highly accurate and effective. The use of invisible near-infrared fluorescent light imaging in swine afflicted with melanoma resulted in SLN identification in a very short time period (1 min), with a success rate of 100%, and suggests the potential for use both in real time to guide surgery and to assist in tissue analysis [164]. Human studies indicate similar levels of efficacy: near-infrared fluorescence also achieved a 100% detection rate in cutaneous melanoma patients, with no reported complications [167].

Image guidance is an effective aid during the surgery and improves surgical outcomes. For nearly all patients, US-guided incisional biopsy resulted in a greater average resected tumor thickness (2.9 mm) compared to non-US-guided incisional biopsy (2.1 mm) [161]. In melanoma patients, IGS improves surgery survival rates and increases the amount of tumor tissue successfully resected [162,165].

IGS also reduces the risk of post-surgical melanoma recurrence. Studies show that human patients with malignant brain melanoma who underwent IGS stayed locally free of melanoma until death [166]. In addition, experiments conducted in mouse models of orthotopic breast cancer and melanoma demonstrated lower recurrence rates in mice that underwent IGS compared to those that underwent non-IGS. Further, for mice that had recurrence after surgery, the recurrence was delayed in IGS mice compared to non-IGS mice. About 10% fewer mice had a recurrence of melanoma after IGS, and recurrence happened approximately 10–20 days later in the IGS group than in the non-IGS group [162,163]. These studies conducted in both human patients and murine models indicate the potential efficacy of IGS for treating melanoma and preventing recurrence; however, additional studies should be conducted to verify these findings in cutaneous melanoma patients.

While IGS most commonly utilizes lymphoscintigraphy, indocyanine green (ICG)-based fluorescence imaging is a possible alternative. A meta-analysis found that there was no difference in diagnostic accuracy between lymphoscintigraphy and fluorescence imaging, suggesting that fluorescence imaging can be used intra-operatively during SLNB procedures [160].

### 6.3. Role of Computer-Assisted Imaging

Computer systems can be used to process clinical imaging and improve interpretation. Computer-based systems typically process images by first reducing them to important features through object segmentation, shape analysis, color and structure analysis, line detection, and texture identification [147,168,169]. After feature extraction, these features can be selected and analyzed to produce the output (e.g., a diagnosis of melanoma or no diagnosis) [148]. This process has limitations: there may be shape irregularities, noise in the image, and other factors that can confound automatic image analyses [147]. Despite these imperfections, feature extraction from images is still robust; using just feature extraction from whole-slide imaging with machine learning allows 96% accuracy in the diagnosis of nevus versus cutaneous malignant melanoma [149].

When computer-assisted imaging techniques are used for diagnostic purposes (computer-assisted diagnosis, or CAD), the diagnostic accuracy is comparable to that of clinicians without computer assistance. One study showed that clinicians had a sensitivity of 98% and specificity of 92% in detecting melanoma, while a computer-based dermatoscopic system (Dermogenius) had a sensitivity of 87% and specificity of 96.5% [146,147]. Overall, CAD’s high sensitivity suggests that it could assist physicians as a “rule-out” diagnostic test for melanoma to minimize false negatives [146]. Given that CAD-assisted diagnosis is supported by 74% of patients (provided that a physician checks and verifies CAD’s results), this is a real and feasible possibility for future clinical practice in melanoma care [150].

### 6.4. Role of AI in Radiographic Imaging and Early Detection

AI can be used with a variety of data types, including image data, and is able to account for multiple types of data within the same model (e.g., imaging, numeric, and categorical). This makes AI a highly comprehensive tool that utilizes many forms of data to improve diagnostic practices. Many studies have shown that AI is able to diagnose skin cancers with greater or comparable accuracy than clinicians (even specialists) [151,152,153,154], though not all models reported in the literature have reached that level of performance [155]. Although one study showed that an AI model led to unnecessary biopsies, physicians assisted by AI have greater diagnostic accuracy than those without [156,157,158,159].

AI usage also offers practical advantages over traditional diagnostic methodologies. For instance, AI diagnostic tools can be offered in the form of smartphone apps without the need for complex medical equipment or physician supervision [151,170,171]. AI could also increase access to care by reducing referrals and patient wait times for diagnosis, thus improving early detection rates [145].

Given how rapidly AI has infiltrated patients’ lives, it seems likely and feasible for AI to be integrated into diagnostic and treatment procedures for melanoma [172,173,174]. Physicians generally rated AI-powered decision-support systems as useful [155,158]. In fact, there is already an FDA-approved AI-enabled skin cancer detection device called DermaSensor [145].

However, patients seem divided when it comes to trusting results from AI, and this may prevent AI from being rapidly integrated into daily practice. Some studies have shown that patients were reluctant to follow recommendations coming from solely AI (though physicians who used AI were perceived as “innovative”) [175], while others showed that patients approved of AI usage by physicians [158,176]. Financial concerns also limit the integration of AI into clinical practice; using an AI-based mobile app resulted in additional costs compared to the current standard of care and increased insurance claims [145,170].

AI, however, is not without several critical limitations. Despite these promising results, several important limitations must be addressed before AI can be widely integrated into melanoma diagnosis and management. Many published AI models have been developed and evaluated using highly curated datasets that may not fully represent the diversity of patients, imaging devices, and lesion subtypes encountered in routine clinical practice [177]. Recent prospective multicenter studies have noted that previous investigations often relied on relatively homogeneous datasets, raising concerns about model generalizability and performance [177]. Furthermore, relatively few AI systems have undergone rigorous prospective external validation, and additional multicenter studies are needed to establish their real-world clinical performance [159]. Using a prospective study design is important because retrospective studies suffer from several limitations, including the selection of only cases with high-quality imaging or many diagnostic features, the Clever Hans bias, and the inclusion of few out-of-distribution cases.

Interpretability also remains an important challenge. Many deep learning algorithms function as “black-box” systems, making it difficult for clinicians to understand the rationale underlying their predictions. This lack of transparency has been identified as a barrier to clinician trust and adoption [178]. Fortunately, some emerging evidence comparing explainable AI with other AI systems for melanoma diagnosis indicates that explainable AI performs just as well as other AI methods. Furthermore, experienced clinicians benefited from explainable AI, while inexperienced ones benefited from regular AI models. This possibly suggests that while AI can offset a lack of melanoma diagnostic experience or knowledge, explainable AI with human-interpretable factors is particularly important for further fine-tuning a well-informed diagnosis and providing additional factors for clinician to consider before making a final decision.

Medico-legal issues are also another barrier to the widespread clinical adoption of AI systems, even those that have already been approved by regulatory bodies such as the FDA. Despite encouragement by the FDA to incorporate autonomous AI into clinical practice, liability is a major concern preventing wide adoption, as it is unclear who will be liable for the inevitable mistakes that fully autonomous AI would make [179]. Fear of medical malpractice claims due to poor AI decision-making is prominent among physicians. However, as more autonomous AI systems are approved, some have even begun to be incorporated into standard care, lowering malpractice liability associated with AI usage.

## 7. Conclusions

**Figure 3:**
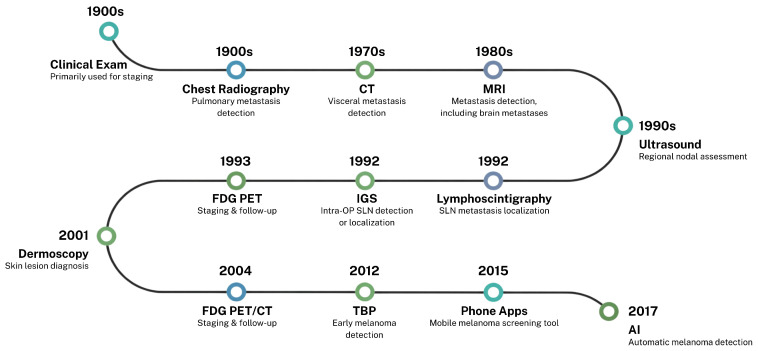
Summary of imaging techniques and their technological applications in chronological order, from the oldest used (top left) to the most recent innovations (bottom right). The years indicated on the timeline are the earliest published report for each imaging technique in melanoma or cancer clinical practice. Note: CT = computed tomography, MRI = magnetic resonance imaging, IGS = image-guided surgery, PET = positron emission tomography, FDG PET = fluorodeoxyglucose positron emission tomography, TBP = total-body photography, AI = artificial intelligence.

This review covered a wide variety of imaging techniques used in melanoma care over the last century, summarized in Figure 3. While some techniques, such as lymphoscintigraphy and MRI, have been used for a long period of time and will likely continue to be used frequently in the future, other techniques, like chest X-ray, are more outdated and may be completely replaced in the near future. New inventions continue to be created in this field, including smartphone apps that provide mobile screening tools and AI-powered detection, which are gradually being incorporated into clinical practice and will shape the future of melanoma diagnosis and treatment.

## Data Availability

No new data were created or analyzed in this study.

## Abbreviations

CXR	Chest X-rays
CT	Computed tomography
MRI	Magnetic resonance imaging
US	Ultrasound
PET	Positron emission tomography
FDG	Fluorodeoxyglucose
SPECT	Single-photon emission computed tomography
LN	Lymph node
SLNB	Sentinel lymph node biopsy
SLN	Sentinel lymph node
PAI	Photoacoustic imaging
MSOT	Multispectral optoacoustic tomography
AJCC	American Joint Committee on Cancer
NCCN	National Comprehensive Cancer Network
ESMO	European Society for Medical Oncology
TBP	Total-body photography
EIS	Electrical impedance spectroscopy
RCM	Reflectance confocal microscopy
IGS	Image-guided surgery
RECIST	Response Evaluation Criteria in Solid Tumors
ctDNA	Circulating tumor deoxyribonucleic acid
AUC	Area under the curve
FNAC	Fine-needle aspiration cytology
EANM	European Association of Nuclear Medicine
FCH	Flurocholine
ICG	Indocyanine green
CAD	Computer-assisted diagnosis
AI	Artificial intelligence
